# Unfavorable efficacy to ^131^I ablation in BRAFV600E mutant papillary thyroid carcinoma with positive TgAb

**DOI:** 10.18632/oncotarget.22129

**Published:** 2017-10-26

**Authors:** Na Zhang, Jun Liang, Yan-Song Lin

**Affiliations:** ^1^ Department of Nuclear Medicine, Peking Union Medical College Hospital, Beijing 100730, China; ^2^ Department of Oncology, Peking University International Hospital, Beijing 102206, China

**Keywords:** papillary thyroid carcinoma, BRAFV600E mutation, TgAb, radioiodine remnant ablation, efficacy

## Abstract

The BRAFV600E mutation has shown a close relationship of aggressiveness in papillary thyroid carcinoma (PTC), while it remains unclear about its influence on the therapeutic response. As a common clinicopathologic risk factor, thyroglobulin antibody (TgAb) may have a correlation of prognosis in PTC. The objective was to investigate the relationship between BRAFV600E mutation and TgAb, and their combined effect on efficacy to radioiodine remnant ablation (RRA). This was a retrospective study including 298 PTC patients and they were divided into four groups according to the combined status. The BRAFV600E mutation rates declined along with increasing TgAb levels in the entire cohort. The ablative efficacy in terms of success or failure rate was statistically different among four groups (89.7%, 74.1%, 67.5%, 57.8%, respectively, *P*=0.009), group with both positive BRAF and TgAb presented the lowest efficacy. The combined status was associated with the poor efficacy to RRA independently (*P*=0.029). Among patients with positive TgAb, the effect of RRA in reducing TgAb level might be weakened in BRAF mutant status. The combined status of BRAFV600E mutation and positive TgAb predicts low efficacy to RRA and might be served as an independent unfavorable prognostic factor for PTC. BRAF mutant might weaken the effect of RRA in reducing TgAb levels in PTCs.

## INTRODUCTION

Papillary thyroid carcinoma (PTC) is the most common malignant thyroid tumor, accounts for 85%-90% of all thyroid cancers, with an increasing incidence in recent decades [1-3]. PTC is a relatively indolent cancer, which usually presents a favorable prognosis with 10-year survival rates over than 90% after appropriate treatment including thyroidectomy, subsequent radioiodine ablation and thyroid stimulating hormone (TSH) suppressive therapy. Up to 25%-30% PTC patients have TgAb-positive test when diagnosed and emerge a higher frequency in younger females [4-6]. The association between TgAb and PTC has been explored by many studies, which TgAb had been demonstrated as an independent predictive factor, and may associated with a higher prevalence of PTC [5, 7]. PTC patients with persistent elevated TgAb levels posttreatment have a higher prevalence for persistent or recurrent disease during subsequent follow-up, and the higher the TgAb, the higher the prevalence, this part of patients warrant closer follow-up than patients who display a falling TgAb trend after therapy [8-10]. However, the mechanism of this adverse prognosis caused by TgAb remains uncertain.

Recently, several oncogenic events have been confirmed to be associated with PTC, among them the BRAFV600E mutation is the frequent genetic alteration, which has evoked great attention and acts as a vital role in the tumorigenesis of PTC through activating the mitogen-activated protein kinase (MAPK) signal pathway [11]. Most studies reported that PTC patients harboring the BRAFV600E mutation show up aggressive characteristics such as bigger tumor size, more advanced tumor stage, extrathyroidal extension, nodal recurrence and radioactive iodine resistance then lead to a poor clinical outcome [12-14]. Although our team did not find a worse clinical response to postsurgical RRA therapy in BRAFV600E mutation PTC patients without distant metastases, but we did find this mutation holds the promise to predict a poorer radioiodine uptake in distant metastases and poorer prognosis [14, 15].

So far, both BRAFV600E mutation and positive TgAb can cause unsatisfied prognosis in PTC have been reported, but the joint effect of the two statuses to RRA has not yet been studied. The present study aims to investigate the impact of BRAFV600E mutation combined with positive TgAb on efficacy to RRA in PTC patients without distant metastases.

## RESULTS

### Characteristics of patients

**Figure 1:**
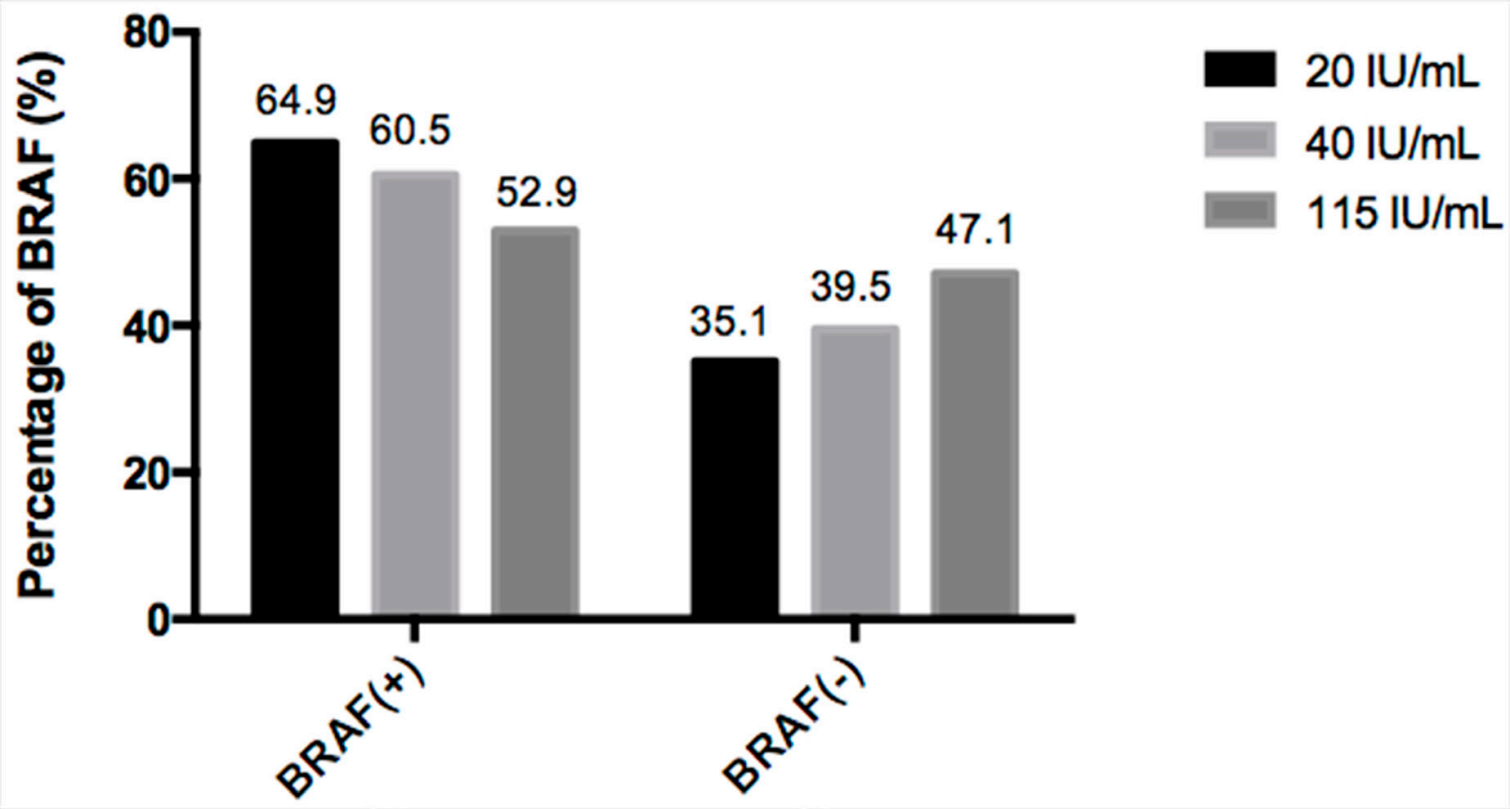
Percentage of BRAF (+/-) with different TgAb concentrations When subdivided the TgAb concentration in 20 IU/mL, 40 IU/mL and 115 IU/mL cases, the rates of BRAFV600E mutation declined along with increasing TgAb concentrations.

A total of 298 patients were included in this study with a male-to-female ratio of 1:2.20 and a mean age of 39.42±11.56 years at diagnosis. The prevalence of BRAFV600E mutation of all patients was 73.5% (219/298) and the TgAb-positive comprised 85 (28.5%) patients with a measurement of TgAb≥115 IU/mL. More males (*P*=0.006), older age (*P*=0.000), more extracapsular invasiveness (*P*=0.014) and advanced TNM stage (*P*=0.006) were more likely to harbor BRAFV600E mutation, indicating BRAFV600E was associated with some aggressive features in PTC. While TgAb positive were more widespread in younger female patients with less bilateral tumour lesions (*P*=0.000, *P*=0.001, *P*=0.009, respectively, Table 1). The BRAFV600E mutation rates tended to decline from 64.9% to 52.9% along with the elevated TgAb concentrations from 20 IU/mL to 115 IU/mL (Figure 1).

**Table 1:** Disease-related characteristics of study cohort

Characteristics	Total	BRAF(+)/BRAF(-)	*P* value	TgAb(+)/TgAb(-)	*P* value
Number	298	219/79		85/213	
Sex, *n* (%)					
Male	93(31.2)	78(35.6)/15(19.0)	0.006*	11(12.9)/82(38.4)	0.000*
Female	205(68.8)	141(64.4)/64(81.0)		74(87.1)/131(61.6)	
Age (years)					
Mean ± SD	39.42±11.56	41.06±11.50/34.86±10.52	0.000*	36.01±12.26/40.78±11.01	0.001*
Tumor size (cm)					
Median (range)	1.0(0.6-1.5)	1.0(0.6-1.5)/1.1(0.6-1.8)	0.590	1.1(0.6-1.8)/1.0(0.6-1.5)	0.170
Tumor multifocality, *n* (%)					
No	130(43.6)	96(43.8)/34(43.0)	0.902	36(42.4)/94(44.1)	0.780
Yes	168(56.4)	123(56.2)/45(57.0)		49(57.6)/119(55.9)	
Tumor lesion, *n* (%)					
Unilateral	194(65.1)	139(63.5)/55(69.6)	0.326	65(76.5)/129(60.6)	0.009*
Bilateral	104(34.9)	80(36.5)/24(30.4)		20(23.5)/84(39.4)	
Extracapsular invasiveness, *n* (%)					
No	131(44.0)	87(39.7)/44(55.7)	0.014*	42(49.4)/89(41.8)	0.231
Yes	167(56.0)	132(60.3)/35(44.3)		43(50.6)/124(58.2)	
TNM-Stage, *n* (%)					
I	194(65.1)	130(59.4)/64(81.0)	0.006*	60(70.6)/134(62.9)	0.462
II	2(0.7)	2(0.9)/0(0)		0(0)/2(0.9)	
III	43(14.4)	36(16.4)/7(8.9)		12(14.1)/31(14.6)	
IV	59(19.8)	51(23.3)/8(10.1)		13(15.3)/46(21.60)	

*P<0.05.

The clinicopathologic features had significant differences in sex (*P*=0.000), age (*P*=0.000) and ps-Tg levels (*P*=0.000) among the four groups, more younger females were prevalent in group 2 contrasts with other groups. The difference regarding ps-Tg levels may attribute to the interference of TgAb. Furthermore, as shown in Table 2, the radioiodine dose presented a downward trend from group 1 to group 4 (*P*=0.000), indicating a more intensive initial radioiodine therapeutic intention to patients of group 1.

**Table 2:** Clinicopathologic features of the four groups

Clinicopathologic features	Group 1 (*n*=45)	Group 2 (*n*=40)	Group 3 (*n*=174)	Group 4 (*n*=39)	Univariate Analysis	
					*F*/*χ*^*2*^/*H*	*P* value
Sex, *n (%)*						
Male	7(15.6)	4(10.0)	71(40.8)	11(28.2)	21.144	0.000*
Female	38(84.4)	36(90.0)	103(59.2)	28(71.8)		
Age (years)						
Mean ± SD	38.36±13.35	33.38±10.46	41.76±10.91	36.38+10.51	7.516	0.000*
Tumor size (cm)						
Median (range)	1.0(0.6-1.5)	1.4(0.7-2.2)	1.0(0.7-1.5)	0.9(0.5-1.5)	7.121	0.068
Tumor multifocality, *n (%)*						
No	19(42.2)	18(45.0)	77(44.3)	17(43.6)	0.081	0.994
Yes	26(57.8)	22(55.0)	97(55.7)	22(56.4)		
Tumor lesion, *n (%)*						
Unilateral	33(73.3)	32(80.0)	106(60.9)	23(59.0)	7.234	0.065
Bilateral	12(26.7)	8(20.0)	68(39.1)	16(41.0)		
Extracapsular invasiveness, *n (%)*						
No	18(40.0)	24((60.0)	69(39.7)	20(51.3)	6.622	0.085
Yes	27(60.0)	16(40.0)	105(60.3)	19(48.7)		
TNM-Stage, *n (%)*						
I	28(62.2)	32(80.0)	103(59.2)	32(82.1)	12.976	0.164
II	0(0)	0(0)	2(1.1)	0(0)		
III	8(17.8)	4(10.0)	28(16.1)	3(7.7)		
IV	9(20.0)	4(10.0)	41(23.6)	4(10.3)		
^131^I dose						
Median (range)	100(30-150)	60(30-150)	30(30-150)	30(30-30)	21.293	0.000*
Ps-Tg level (ng/mL)						
Median (range)	0.1(0.1-1.5)	0.3(0.1-1.9)	2.1(0.6-7.0)	1.9(0.7-6.3)	35.987	0.000*
Ps-TSH level (μIU/mL)						
Median (range)	100.0(73.2-138.4)	130.5(86.5-150.0)	100.0(75.7-129.3)	103.3(75.3-137.5)	6.520	0.089

*P<0.05; Ps-Tg, preablative-stimulated thyroglobulin; Ps-TSH: preablative thyroid stimulating hormone.

### Association between RRA efficacy and different combination of TgAb and BRAFV600E

**Figure 2:**
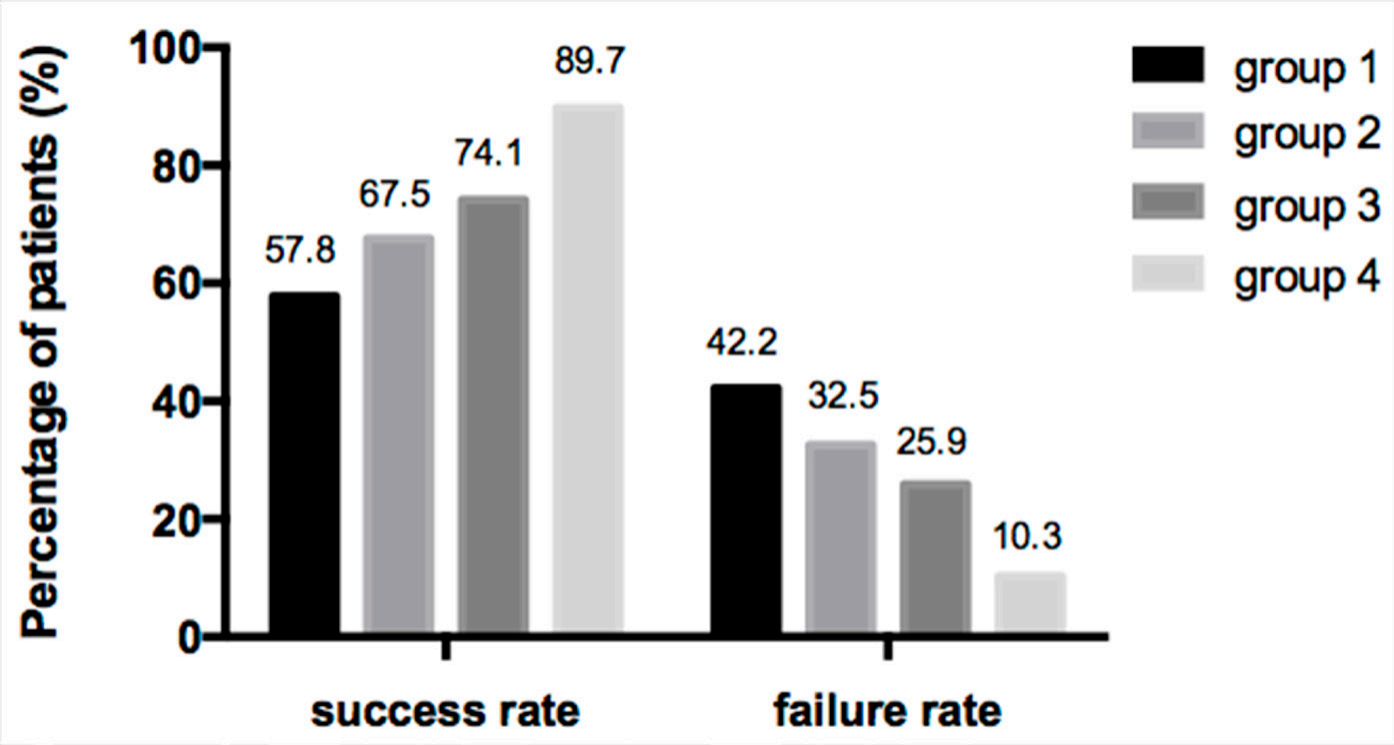
Percentage of patients with different efficacy to RRA in four groups The efficacy in terms of RRA success rates were statistically different among four groups and presented a significant decreasing trend from group 4 to group 1 (89.7%, 74.1%, 67.5%, 57.8%, respectively, *P*=0.009).

The efficacy in terms of RRA success rates were statistically different among four groups and presented a significant decreasing trend from group 4 to group 1 (89.7%, 74.1%, 67.5%, 57.8%, respectively, *P*=0.009, Figure 2), the RRA success rate with coexisting BRAFV600E mutation and positive TgAb (group 1) was the lowest and also significantly lower than that associated with either status alone (group 2 and group 3), whereas, patients with both negative BRAFV600E mutation and TgAb (group 4) presented the highest success rate. The results of the multivariate analysis are shown in Table 3. The efficacy to RRA was not significantly associated with TgAb (+), BRAF(+), sex, age, ^131^I dose, ps-Tg level except the coexisting status of BRAFV600E mutation and positive TgAb (OR, 2.121; 95% CI=1.081-4.161; *P*=0.029), the coexisting status was confirmed to be independent predictive factor for efficacy to RRA.

**Table 3:** Multivariate analysis of RRA efficacy according to clinicopathologic factors

Variables	Odds ratio	95%CI		*P* value
		Lower	Upper	
TgAb(+)+BRAF(+)	2.121	1.081	4.161	0.029*
TgAb(+) only	1.205	0.562	2.582	0.632
BRAF(+) only	1.579	0.821	3.038	0.171
Sex	0.848	0.476	1.514	0.578
Age	0.996	0.974	1.018	0.719
^131^I dose	0.999	0.995	1.002	0.440
Ps-Tg level	0.997	0.992	1.003	0.329

*P<0.05; CI, confidence interval; Ps-Tg, preablative-stimulated thyroglobulin.

### Change of TgAb level after RRA in positive TgAb patients

**Figure 3:**
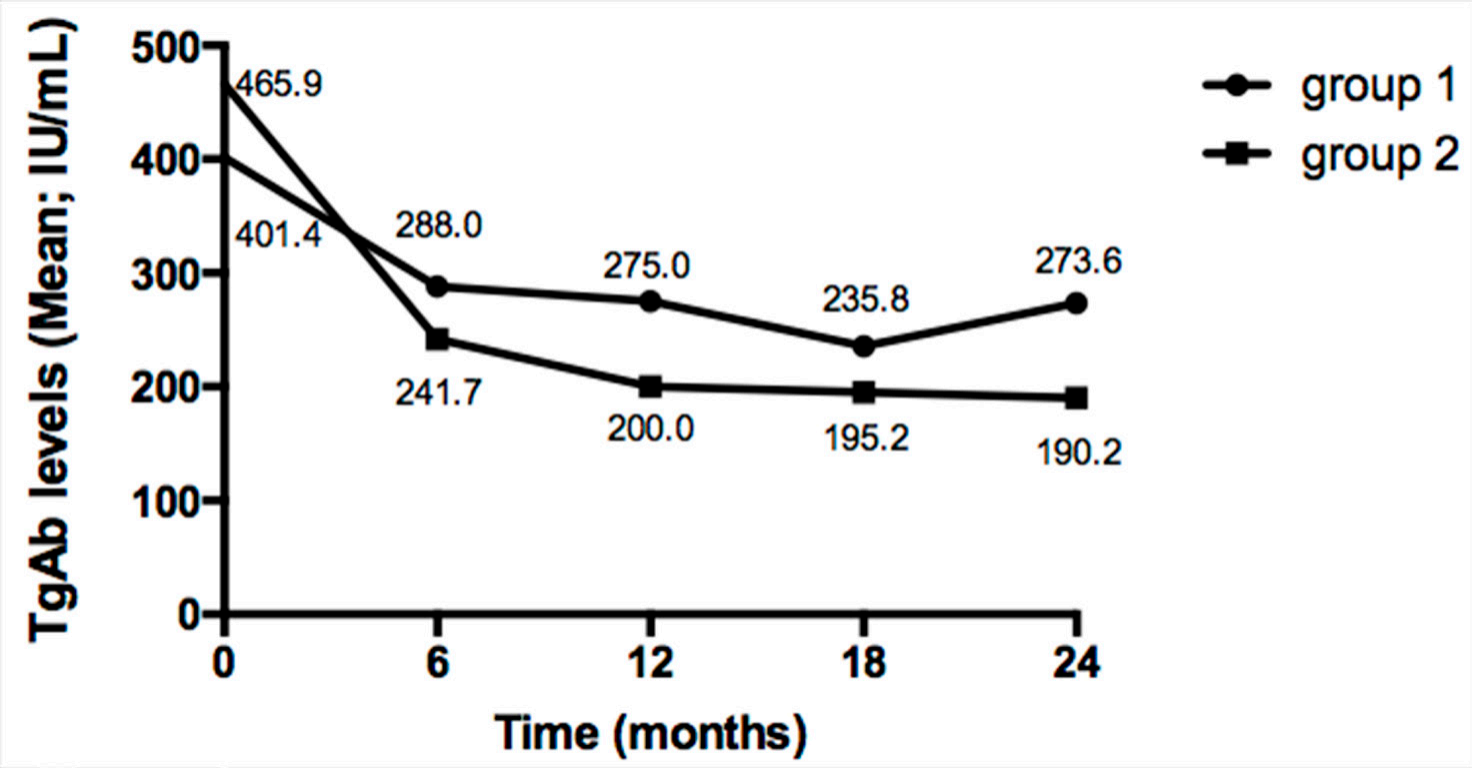
Change of TgAb levels after RRA in positive TgAb patients TgAb levels in group 1 were initially slightly lower, however, gradually higher compared with TgAb levels in group 2 during the follow-up period though the differences were without statistical significance (all *P*>0.05). Furthermore, TgAb levels in group 1 appeared an uptrend from 18 months to 24 months while TgAb levels in group 2 decreased sequentially.

With a purpose to discover the changing pattern of TgAb levels in patients with positive TgAb after the initial RRA, we followed up the 85 positive TgAb patients (group 1 and group 2) from the time of RRA to 24 months thereafter, TgAb levels were measured regularly every 6 months. As shown in Figure 3, TgAb levels in BRAF mutant group (group 1) were initially slightly lower (mean, 401.4 IU/mL vs 465.9 IU/mL), however, gradually higher compared with TgAb levels in BRAF wild group (group 2) during the subsequent 24 months of follow-up though the differences were without statistical significance (all *P*>0.05). Furthermore, TgAb levels in BRAF mutant patients appeared an uptrend from 18 months to 24 months while TgAb levels in BRAFV600E wild patients decreased sequentially.

## DISCUSSION

As one of the initial managements of PTC, RRA plays a vital role in reducing the recurrence rates and prolonging survival in intermediate to high risk patients [17]. Several factors have been demonstrated that weakening the efficacy of RRA including surgical approach, local or distant metastases, sodium-iodide symporter (NIS), BRAFV600E mutation and serum TgAb levels [13, 18-21]. Among them, BRAF mutation has been widely explored recently. In a multicenter study of 2,099 patients, BRAFV600E mutation was associated with PTC recurrence (*P*<0.001) even after RRA. The authors also explored the relationship between BRAFV600E mutation and various characteristics, such as age, lymph node involvement, but TgAb was not evaluated within, which is a common clinicopathologic risk factor with a frequency range from 25% to 30% in PTC patients [4, 13]. In 2017, Li et al. [22] investigated the link between TgAb titers and PTC aggressiveness, they mentioned patients with positive TgAb were more likely to harbor BRAFV600E mutation and NF-κB expression, moreover, those with positive TgAb tended to have poor clinical outcome after RRA, however, no further exploration on the joint effect of BRAFV600E mutation and positive TgAb to RRA in their study. So far, the coexisting effect of the two statuses on efficacy to RRA in PTC patients remains unclear.

In our study, the frequency of BRAFV600E mutation and positive TgAb were 73.49% and 28.52% respectively, which were comparable to other studies [4, 23]. As found in most studies [12-14], our data also revealed that PTC patients harboring BRAFV600E mutation presented with older age, more males, extracapsular invasiveness and advanced TNM stage as manifestations of stronger aggressiveness. Of note, when subdivided the TgAb titers, our study showed that BRAFV600E mutation, as an aggressive factor, declined in the incidence with increasing TgAb levels, which was inconsistent to Li’s findings [22]. What factor could be the causation of the differences between the two studies, we noticed that there were only 10 TgAb-positive cases (10/113) in the aforementioned study with only one cutoff value (40 IU/mL), the limited samples and cutoff value may somewhat less representative and persuasive. TgAb is considered as a serologic marker of autoimmune thyroid disease (AITD), which involved in PTC pathogenesis [24]. Recently, a large retrospective survey indicated the beneficial effect of autoimmunity on thyroid cancer, PTC with Hashimoto’s thyroiditis (HT) presented with less aggressive clinical stage, such as younger age, early TNM stage and smaller tumor size [25]. Kim et al. [1] reported the BRAFV600E mutation, as one of aggressive molecular events, was detected at a lower frequency in PTC patients with coexistent HT (24.4% vs 14.5%, non-HT vs HT, *P*<0.05). Given the evidences mentioned above, and findings herein that more younger females, less bilateral lesions and BRAFV600E mutation among TgAb positives, it seemed that PTC with positive TgAb behaves relative indolent. Thus autoimmunity may be involved in PTC pathogenesis in a less invasive way, since BRAFV600E as an aggressive event is not dominant therein.

In order to explore the combined effect on the efficacy to RRA, in this study, for the first time, we divided patients into four groups according to the combined two statuses. It is noteworthy the success rates to RRA presented a significant decreasing trend from group with both negative TgAb and BRAF mutation to group with both positive status (89.7%, 74.1%, 67.5%, 57.8%, respectively, *P*=0.009), the RRA success rate with coexisting BRAFV600E mutation and positive TgAb was also significantly lower than that associated with either status alone, implying an synergistic and incremental effect of the coexisting two statuses, thus attenuate the efficacy to RRA. One possible explanation for this strong cooperative effect of the combined status on the efficacy may be related to that both them are associated with lower expression levels of NIS, directly or indirectly, which, reduces the therapeutic efficacy to RRA mainly through mediating the process of tumor cells incorporating radioiodine [26-30]. In addition, we noticed that there were statistically significant differences among the four groups regarding sex, age, ^131^I dose and ps-Tg level. In order to further identify the independent factors associated with the efficacy to RRA, we following conducted a multivariate analysis, and importantly we found that, the combined status was the only contributor independently influencing the efficacy to RRA. Thus, in this paper, we have identified a novel background-coexistence of BRAFV600E mutation and positive TgAb-which forebodes a poorer efficacy to RRA of PTCs independently, meanwhile, provides unfavorable prognosis information even before RRA.

Serum Tg measurement is important for follow-up after RRA in PTCs, however, the accuracy of Tg measurement is interfered by TgAb, especially at high titers, limiting the predictive value of Tg [9, 31]. Thus, the follow-up of PTCs with positive TgAb remains a clinical puzzlement. When TgAb is positive, successful RRA would be defined primarily by negative imaging findings, however, TgAb levels will change after treatment and the disappearance maybe up to 10 months according to our previous studies [32]. Therefore, based upon the evidences from some researches that positive TgAb during follow-up indicating persistent disease [4, 31], the 2015 ATA guidelines proposed that TgAb could also serve as a surrogate tumor marker [16]. Hence, in this study, the changing pattern of TgAb levels in patients with positive TgAb was analyzed separately. Comparing with TgAb levels in BRAF wild group, TgAb levels in BRAF mutant group were initially lower, then, gradually higher during the subsequent 24 months of follow-up, even after administering a higher radioiodine dose, suggesting a weaken role of RRA in reducing TgAb in BRAF mutant status. So it seemed that patients with coexisting background carrying a poorer serological response in contrast to patients with positive TgAb only.

In conclusion, a novel background-coexistence of BRAFV600E mutation and positive TgAb was investigated in this study, suggesting a poorer efficacy to RRA of PTCs independently and conveying unfavorable prognosis information. The rates of BRAFV600E mutation declined along with rising TgAb levels. BRAF mutant might weaken the effect of RRA in reducing TgAb level in PTC patients.

## MATERIALS AND METHODS

### Patients and study design

A total of 1,254 patients with differentiated thyroid carcinoma (DTC) were enrolled in this retrospective study and had undergone total or near-total thyroidectomy, subsequent RRA and TSH suppressive therapy in our hospital from June 2012 to June 2016. The exclusion criteria were: (1) pathological types other than PTC, (2) lack of BRAFV600E analysis result or TgAb test result, (3) with distant metastases suspected by elevated serum thyroglobulin (Tg) level or imaging examination results including diagnostic whole body scan (Dx-WBS), chest CT, PET/CT or histopathological biopsy, (4) follow-up time less than 6 months. TgAb was considered “negative” or “positive” based on the specific assay cutoff value in our hospital (negative<115IU/ml; positive≥115IU/ml). In the end, 298 patients were included in our study and were divided into four groups according to the combined status: group 1 (both positive in TgAb and BRAFV600E, *n*=45); group 2 (TgAb positive only, *n*=40); group 3 (BRAFV600E mutant only, *n*=174); group 4 (both negative, *n*=39). To observe the relationship between BRAFV600E mutation and TgAb concentration, we further investigated the mutation rate in three cases: TgAb levels were higher than 20 IU/mL, 40 IU/mL, 115 IU/mL, respectively.

All patients underwent RRA after preparation of levothyroxine (L-T4) withdrawal together with a strict low-iodine diet for at least 2 weeks, ablative doses of ^131^I (1.11-5.55 GBq, 30-150 mCi) were administered to all patients according to the comprehensive assessment of postoperative pathology and examinations before RRA. L-T4 was administered at 3 days and the posttreatment whole body scan (Rx-WBS) was performed at 7 days after the radioiodine administration. At 6 months after RRA all patients were informed to withdraw L-T4 until TSH levels above 30 μIU/mL, measured serum TgAb levels, Tg levels, performed Dx-WBS and other imaging examinations. Patients with positive TgAb before RRA were followed up to 24 months for observe the changing in TgAb levels.

The efficacy to RRA in PTC was divided into success or failure according to the 2015 American Thyroid Association (ATA) guidelines [16], when the TgAb is present, the success of RRA was defined as follows: (1): Dx-WBS demonstrated no residual thyroid tissue or diffuse radioiodine uptake in bone or lung at 6 months after the initial radioiodine treatment; (2) no recurrence or metastases suspected by other imaging examinations (chest CT, PET/CT, bone scintigraphy, thyroid ultrasound).

### BRAFV600E mutation analysis

A consecutive 4-μm-thick section was taken from the paraffin blocks of primary tumor for DNA extraction, then using a commercially available kit (QIAamp DNA FFPE tissue kit 50, United Kingdom) to extract DNA. BRAF mutation real-time fluorescence testing kit (ADx-ARMS, AmoyDX, Xiamen, China) was used to conduct the quantitative real-time polymerase chain reaction and the kit include Taq DNA polymerase, oligonucleotide primers, nucleotides, hydrolysis oligonucleotide probes and buffers. The extracted 5-μL DNA was mixed with 0.4μL of Taq DNA polymerase and 35 μL of other reagents in the kit. All the above reactions were performed in a qRT-PCR machine (ABI 7500, Applied Biosystems, Foster City, CA) as follows: denaturation 5 min at 95°C, followed by annealing 25 s at 95°C, 20s at 64°C and 20s at 72°C. The above procedures were repeated for 15 cycles and extended for 31 cycles of 25s at 93°C, 35s at 60°C, 20s at 72°C, Fluorescence increased with exponential growth of the PCR products geometrically, which was used to judge the threshold cycle (CT). Threshold cycle less than 28 is defined as positive, otherwise negative.

### Serological test

Serological examinations including TSH, Tg and TgAb were measured before RRA. TSH level was determined by chemiluminescence immunoassay (Siemens Healthcare Diagnostics Inc, New York, NY), the measurement was 0.004 to 150 μIU/mL. Tg and TgAb levels were determined by electrochemiluminescence immunoassay (Roche Diagnostics GmbH, Germany), with the measurement range from 0.100 to 500 ng/mL and 10 to 4,000 IU/mL, respectively.

### Statistical analysis

Statistical analyses were performed using SPSS (version 22.0; IBM Corp, NY). The associations between BRAFV600E, positive TgAb and characteristics were evaluated with Student’s *t* test, chi-square test and Mann-Whitney *U* test. Analysis of variance (ANOVA) was used to compare age among the four groups. Chi-square test and Kruskal-Wallis rank-sum test were applied to contrast other clinicopathologic features of the four groups. RRA efficacy in four groups was assessed by chi-square test. A multivariate logistic regression was performed to identify the independent factors associated with the efficacy to RRA. *P*<0.05 was considered as statistical significance. All figures were created by GraphPad Prism 6 (Emerald Biotech Co., Ltd, Hangzhou, China).
